# Tumor containing fragment number influences immunohistochemistry positive rate of HER2 in biopsy specimens of gastric cancer

**DOI:** 10.1186/s13000-017-0616-5

**Published:** 2017-05-26

**Authors:** Chen Xu, Yalan Liu, Xiaowen Ge, Dongxian Jiang, Ying Zhang, Yuan Ji, Jun Hou, Jie Huang, Jieakesu Su, Haiying Zeng, Jing Qin, Yingyong Hou

**Affiliations:** 1 Department of Pathology, Zhongshan Hospital, Fudan University, Shanghai, 20032 China; 2 Department of Pathology, School of Basic Medical Sciences & Zhongshan Hospital, Fudan University, Shanghai, 200032 China; 3 Department of General Surgery, Zhongshan Hospital, Fudan University, Shanghai, 20032 China

**Keywords:** Gastric cancer, HER2, Biopsy, Immunohistochemistry

## Abstract

**Background:**

HER2 assessment in biopsy specimens of gastric cancer (GC) is challenging because of the intratumoral heterogeneity. False negative results may be get because of limited biopsy material. The aim of this study is to explore how tumor-containing fragment number and biopsy specimen number affect HER2 immunohistochemistry (IHC) positive rate.

**Methods:**

Eight hundred and ninety biopsy specimens and 459 paired resected specimens were collected. IHC staining of HER2 was performed. HER2 IHC positive (scored 3+) rate was compared based on tumor-containing fragment number, biopsy specimen number, average size and tumor tissue proportion of tumor-containing fragments. The positive predictability of biopsy specimens to resected specimens was analyzed based on tumor fragment number.

**Results:**

HER2 IHC positive rates were 2.0, 3.5, 7.0, 13.2, 17.1, and 15.9% when tumor fragment numbers were 1, 2, 3, 4, 5 and 6 respectively. The rate rose with the increase of tumor fragment number (*P* = 0.004). ROC curve analysis showed that biopsy specimens exhibited positive predictability when tumor fragment number reached 3, but showed better performance when the number was ≥4 (*P* < 0.05). After fragment number reached 4, no statistic differences were reached in either HER2 IHC positive rate or positive predictability with further increase of the number (*P* > 0.05). HER2 IHC positive rate was not associated with biopsy number (*P* = 0.127), average size of tumor fragments (*P* = 0.397), and tumor tissue proportion of tumor fragments (*P* = 0.825) directly.

**Conclusions:**

The number of tumor-containing fragments influences HER2 IHC positive (scored 3+) rate. Greater than or equal to 4 (≥4) tumor fragments give better results in the positive rate as well as positive predictability. We recommend the number of tumor containing fragments be described in the HER2 IHC pathology reports for clinical reference in endoscopic biopsy specimens of GC.

## Background

Accurate assessment of human epidermal growth factor receptor 2 (HER2) is a pivotal issue in gastric cancer (GC) since the Trastuzumab for Gastric Cancer (ToGA) trial proved the value of the targeted therapy in HER2 positive GC patients [1]. In GC, both biopsy and resected specimens are suitable for HER2 analysis. Many GC patients are with inoperable lesions and endoscopic biopsy becomes the only available approach to obtain tumor tissues for HER2 assessment. Unlike resected specimens, biopsy specimens are with more influence factors and in turn more difficult to manipulate [2]. Therefore, it is of clinical importance to explore influential factors and optimize HER2 detection in biopsy specimens.

Several approaches are available for HER2 status assessment, including immunohistochemistry (IHC), fluorescence in situ hybridization (FISH), and silver in situ hybridization (SISH). Among them, IHC represents an effective and robust test that can be used for most specimens [3], and has been proved to be a valuable approach in reflecting HER2 status in GC [1, 4].

Assessment of HER2 status in GC is challenging because of the protein’s affinity for being heterogeneously expressed [4–6]. For endoscopic biopsy specimens, it is a far more serious issue in that biopsy specimens are less manageable than resected specimens in HER2 assessment due to unpredictable tumor tissue amount. The heterogeneity may easily lead to false-negative results in cases with limited biopsy material, suggesting the necessity of extensive tissue sampling [2, 7]. However, no formal consensus related to the number of endoscopic biopsies required for HER2 testing has been widely accepted yet. Several existing guidelines provide vague and discordant recommendations on biopsy number. The NCCN guideline recommended that multiple biopsies (8–10 spots) should be carried out to provide adequate-sized material for histologic interpretation [8]. Ruschoff et al. [9]. have recommended 6 to 8 biopsy fragments in GC for HER2 testing while Kim et al. [10]. have proposed 4 to 6 as acceptable. Recently, several studies aimed to find out the ideal biopsy number for HER2 test in GC and rendered inconsistent numbers including 4 and 5 [11–13].

From all these available studies, we already know that inadequate biopsy materials would lead to less accurate HER2 results. How the biopsy number affects HER2 results and the associations between them are still to be elucidated. Thus in this study, we retrospectively assessed HER2 IHC status in 890 GC biopsy specimens and 459 paired resected specimens, to explore the influences of biopsy number and tumor-containing fragment number on HER2 IHC positive (scored 3+) rate and predictability of biopsy specimens. Besides, other two factors related to tumor tissue amount including average size and tumor tissue proportion of tumor-containing fragments were also subjected to the assessment. We hope that the findings in this work will be beneficial to clinical practice, particularly when only endoscopic biopsy samples are available due to inoperability, which is frequently encountered in GC.

## Methods

### Patients and clinicopathological information collection

The study protocol was approved by the ethics board of Zhongshan Hospital, Fudan University, Shanghai, China. A total of 890 patients were collected. All these patients were diagnosed gastric adenocarcinoma by endoscopic biopsy specimens during March 2013 to January 2014 in Zhongshan Hospital, Fudan University. Only patients with primary tumors were selected for this study. Recurrent tumors, rare histological variants including adenosquamous carcinoma, squamous carcinoma, hepatoid adenocarcinoma, and carcinoma with lymphoid stroma, as well as neuroendocrine tumors were excluded. Within the 890 patients, surgical specimens of 459 cases were available, and HER2 status of paired biopsy and resected specimens were analyzed.

The tumor location was recorded according to the three gastric regions [14], including the upper third, the middle third and the lower third. If more than one part was involved, the location was recorded as “Others”.

The numbers of biopsy specimens and tumor-containing fragments were recorded. A tumor-containing fragment (tumor fragment) referred to a piece of tissue containing 10 or more viable tumor cells in an endoscopic biopsy specimen as previously described [15].

The maximum diameter of each tumor fragment was obtained. Average size of tumor fragments was calculated in each case. The proportion of tumor tissue in tumor fragments of each case was also evaluated and obtained. All the measurements were done with an Aperio AT2 digital slide scanner (Leika Biosystems).

### Immunohistochemistry and pathological assessment

A HER-2/neu (4B5) rabbit monoclonal antibody (Ventana Medical Systems, Inc., Tucson, AZ) was used, and the IHC staining was performed in all the cases with iView DAB Detection Kit (Ventana, Tucson, AZ) on a BenchMark XT automated stainer (Ventana Medical Systems, Inc., Tucson, AZ), following the procedures previous described [16]. For each test, small pieces of GC tissue in which HER2 was scored as 3+ and 0 were used in the same slide as positive and negative controls, respectively. For resected specimens, blocks with tumor tissues as well as adjacent normal tissues were chosen for HER2 analysis, so the normal tissues could work as inner controls.

Hematoxylin and eosin (HE) sections of all the cases were reviewed by two experienced gastrointestinal pathologists to confirm the diagnosis. Tumor differentiation and Lauren classification were evaluated according to the WHO classification of tumors of the digestive system (4^th^ edition) [17].

The HER2 status was assessed by 2 independent observers. If there was any discrepancy, the HER2 status was verified by a discussion panel consisting of 3 observers. All observers were blinded with regard to patient clinicopathological characteristics. HER2 was scored according to the established IHC criteria for GC [9, 10, 18]. Briefly, for biopsy specimens, no reactivity or no membranous reactivity in any tumor cell was defined as 0; tumor cell cluster (≥5 cells) with a faint/barely perceptible membranous reactivity irrespective of percentage of tumor cells stained was defined as 1+; tumor cell cluster (≥5 cells) with a weak to moderate complete, basolateral, or lateral membranous reactivity irrespective of percentage of tumor cells stained was defined as 2+; tumor cell cluster (≥5 cells) with a strong complete, basolateral, or lateral membranous reactivity irrespective of percentage of tumor cells stained was defined as 3+. For resected specimens, no staining or less than 10% tumor cell positive staining was defined as 0; faintly or barely perceptible staining on ≥ 10% tumor cell membrane and only on part of the membrane was defined as 1+; Weak to moderate complete, basolateral, or lateral membranous reactivity in ≥10% of tumor cells was defined as 2+; and strong complete, basolateral, or lateral membranous reactivity in ≥10% of tumor cells was defined as 3+. For HER2 IHC positive (scored 3+) cases, the number of HER2 3+ tumor-containing fragments was also recorded.

### Statistics

*χ*2 test was used for univariate analysis; cross-tabulations with qualitative variables were analyzed with the Pearson *χ*2 test. One way ANOVA was used for the comparison of means among groups. ROC curves were constructed to evaluate the predictive ability of biopsy specimens for HER2 status. Z test was conducted for the comparison of AUC value between ROC curves. *P* value <0.05 was defined as statistically significant. No adjustments were made. All analyses were performed using the statistical package SPSS version 22.0 (SPSS, Inc, an IBM Company, Chicago, IL).

## Results

### Characteristics of the patients

The clinicopathologic characteristics of the 890 patients were shown in Table 1. Patient age ranged from 19 to 92 years with an average of 61.8. The median age was 62 years old. There were 617 male and 273 female with a male to female ratio of 2.26:1. The lower third of the stomach was most frequently affected (409 cases, 46.0%). Poorly differentiated adenocarcinomas accounted for the majority (67.9%), followed by moderate differentiated (31.8%) and well differentiated (0.3%) tumors respectively. As to Lauren classification, the majority were classified as intestinal subtype (50.3%), the rest were classified as diffused type (28.9%), mixed type (19.8%), and indeterminate type (1.0%) respectively.

**Table 1:** Clinicopathologic features of the patients

Clinicopathologic features	Number of cases (%)
Gender	
Male	617 (69.3)
Female	273 (30.7)
Location	
Upper third	213 (23.9)
Middle third	262 (29.4)
Lower third	409 (46.0)
Others	6 (0.7)
Differentiation	
Well	3 (0.3)
Moderate	283 (31.8)
Poorly	604 (67.9)
Lauren Classification	
Intestinal	448 (50.3)
Diffuse	257 (28.9)
Mixed	176 (19.8)
Indeterminate	9 (1.0)
Number of biopsy specimens	
1	7 (0.8)
2	20 (2.2)
3	27 (3)
4	209 (23.5)
5	127 (14.3)
6	433 (48.7)
7	29 (3.3)
8	33 (3.7)
9	0 (0)
10	5 (0.6)
Number of tumor fragments	
1	51 (5.7)
2	85 (9.6)
3	171 (19.2)
4	348 (39.1)
5	117 (13.1)
6	113 (12.7)
7	0 (0)
8	4 (0.4)
9	0 (0)
10	1 (0.1)
HER2 status	
0	240 (27.0)
1+	289 (32.5)
2+	261 (29.3)
3+	100 (11.2)

The number of biopsy specimens ranged from 1 to 10 (without 9). The mean and median biopsy number were 5.30 and 6 respectively. The number of tumor-containing fragments varied from 1 to 10 (without 7 and 9), with a mean number of 3.86 and a median number of 4. The distribution of patients based on biopsy specimen number and tumor fragment number was shown in Table 1.

The average size of tumor fragments ranged from 0.075 cm to 0.352 cm, with a mean and a median of 0.196 cm and 0.197 cm respectively. The tumor tissue proportion of tumor fragments ranged from 11.2% to 93.4% with a mean and a median of 52.4 and 54.3% respectively.

To exclude the disturbance of other factors which may affect HER2 positivity, we compared Lauren subtype, tumor location, average size and tumor tissue proportion of tumor fragments among subgroups divided based on the numbers of biopsy specimens and tumor fragments. These parameters showed no significant differences among the subgroups (Table 2).

**Table 2:** Comparison of Lauren subtypes, tumor location, average size and tumor tissue proportion of tumor fragments based on biopsy number and tumor fragment number

	Lauren subtypes, n (%)				*P* value	Tumor location, n (%)				*P* value	Average size (mean ± SD, cm)	*P* value	Tumor tissue proportion (%)	*P* value
	Intestinal	Diffuse	Mixed	Indeterminate		Upper third	Middle third	Lower third	Others					
Number of biopsy specimens					0.112					0.445		0.977		0.204
1	6 (85.7)	1 (14.3)	0 (0.0)	0 (0.0)		2 (28.6)	1 (14.3)	4 (57.1)	0 (0.0)		0.194 ± 0.012		54.4 ± 10.9	
2	11 (55.0)	6 (30.0)	1 (5.0)	2 (10.0)		5 (25.0)	6 (30.0)	9 (45.0)	0 (0.0)		0.201 ± 0.056		56.7 ± 16.1	
3	12 (44.4)	8 (29.6)	7 (25.9)	0 (0.0)		10 (37.0)	5 (18.5)	11 (40.7)	1 (3.7)		0.192 ± 0.046		52.4 ± 15.6	
4	107 (51.2)	56 (26.8)	43 (20.6)	3 (1.4)		58 (27.8)	53 (25.4)	97 (46.4)	1 (0.5)		0.199 ± 0.038		51.0 ± 17.1	
5	68 (53.5)	33 (26.0)	26 (20.5)	0 (0.0)		39 (30.7)	37 (29.1)	51 (40.2)	0 (0.0)		0.195 ± 0.035		51.8 ± 17.9	
6	215 (49.7)	131 (30.3)	84 (19.4)	3 (0.7)		87 (20.1)	137 (31.6)	205 (47.3)	4 (0.9)		0.196 ± 0.041		53.4 ± 18,1	
7	12 (41.4)	9 (31.0)	7 (24.1)	1 (3.4)		6 (20.7)	7 (24.1)	16 (55.2)	0 (0.0)		0.193 ± 0.043		52.6 ± 18.9	
8	15 (45.5)	10 (30.3)	8 (24.2)	0 (0.0)		6 (18.2)	14 (42.4)	13 (39.4)	0 (0.0)		0.200 ± 0.039		48.5 ± 19.3	
10	2 (40.0)	3 (60.0)	0 (0.0)	0 (0.0)		0 (0.0)	2 (40.4)	3 (60.0)	0 (0.0)		0.202 ± 0.040		43.0 ± 15.7	
Number of tumor fragments					0.126					0.745		0.189		0.530
1	31 (60.8)	14 (27.5)	5 (9.8)	1 (2.0)		10 (19.6)	11 (21.6)	30 (58.8)	0 (0.0)		0.209 ± 0.042		48.8 ± 20.4	
2	46 (54.1)	25 (29.4)	11 (12.9)	3 (3.5)		15 (17.6)	29 (34.1)	41 (48.2)	0 (0.0)		0.190 ± 0.032		50.5 ± 18.9	
3	91 (53.2)	50 (29.2)	29 (17.0)	1 (0.6)		42 (24.6)	49 (28.7)	79 (46.2)	1 (0.6)		0.195 ± 0.039		50.2 ± 17.3	
4	184 (52.9)	89 (25.6)	73 (21.0)	2 (0.6)		92 (26.4)	105 (30.2)	148 (42.5)	3 (0.9)		0.197 ± 0.037		53.8 ± 17.0	
5	48 (41.0)	39 (33.6)	29 (23.9)	1 (0.9)		26 (22.2)	37 (31.6)	54 (46.2)	0 (0.0)		0.193 ± 0.040		52.2 ± 18.0	
6	47 (41.6)	38 (33.6)	27 (23.9)	1 (0.9)		28 (24.8)	29 (25.7)	54 (47.8)	2 (1.8)		0.199 ± 0.041		54.7 ± 17.7	
8	1 (25.0)	1 (25.0)	2 (50.0)	0 (0.0)		0 (0.0)	1 (25.0)	3 (75.0)	0 (0.0)		0.214 ± 0.026		52.5 ± 19.3	
10	0 (0.0)	1 (100.0)	0 (0.0)	0 (0.0)		0 (0.0)	1 (100.0)	0 (0.0)	0 (0.0)		0.204		51.3	

“n” refers to the number of cases. “Average size” refers to average size of tumor-containing fragments. “Tumor tissue proportion” refers to tumor tissue proportion of tumor-containing fragments

### HER2 status and intratumoral heterogeneity

Totally, one hundred patients (11.2%) were classified as HER2 IHC positive (scored 3+). 29.3% of total patients (261 cases) were HER2 equivocal (scored 2+). The rest 529 cases were classified as HER2 IHC negative (59.5%), including 289 cases scored 1+ (32.5%) and 240 cases scored 0 (27.0%). In the HER2 IHC positive (scored 3+) patients, 74 cases were intestinal type GCs (74.0%), 10 were diffuse type GCs (10.0%), and the rest 16 belonged to mixed type (16.0%).

**Fig. 1:**
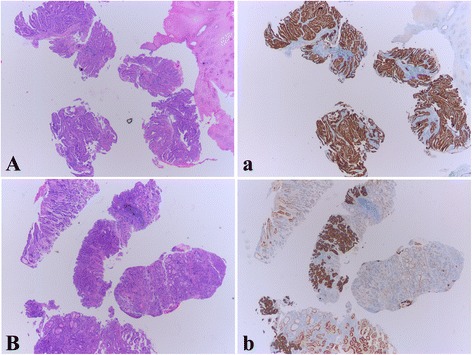
Examples of Intratumoral homogeneity and heterogeneity of HER2 IHC staining in biopsy specimens. **A** The biopsy of an intestinal type GC shows 4 tumor-containing fragments in the 5 biopsy specimens. **a** HER2 IHC staining shows that all the 4 tumor fragments are uniformly 3+ (homogeneous). **B** Four tumor fragments are found in this biopsy of an intestinal type GC. **b** Within the 4 fragments, 3 are stained 3+, the 4^th^ fragment is stained 1+ (heterogeneous). In addition, within the 3 HER2 3+ fragments, one of them demonstrates focally positive

Within the HER2 IHC positive (scored 3+) patients, 47 patients (47.0%) were regarded as homogenous since HER2 staining were uniformly strong in all the tumor-containing fragments (Fig. 1A, a). The left 53 cases (53.0%) were defined as intratumorally heterogeneous because only part of tumor fragments were HER2 IHC positive staining (Fig. 1B, b). The number of HER2 IHC positive (scored 3+) fragments ranged from 1 to 6. The distribution of patients based on the number of HER2 IHC positive fragments was shown in Table 3.

**Table 3:** Tumor-containing fragment number and HER2-positive fragment number in HER2 IHC positive patients

Number of tumor fragments	Total	Number of HER2 3+ fragments	n
1	1	1	1
2	3	1	2
		2	1
3	12	1	1
		2	2
		3	9
4	46	1	7
		2	6
		3	10
		4	23
5	20	1	0
		2	6
		3	5
		4	1
		5	8
6	18	1	2
		2	2
		3	4
		4	4
		5	1
		6	5

“Total” refers to the total number of HER2 positive (scored 3+) cases with different number of tumor-containing fragments

“n” refers to the number of cases with different number of HER2 positive (scored 3+), fragments

Compared with the diffused type (3 homogeneously HER2 IHC positive cases (30.0%) and 7 heterogeneously positive cases (70.0%)) and the mixed type (5 homogeneous cases (31.3%) and 11 heterogeneous cases (68.7%)), the intestinal type demonstrated a smaller proportion of heterogeneous cases (35 cases, 47.3%) and a larger proportion of homogeneous cases (39 cases, 52.7%) . But the differences did not reach statistical significance (*P* = 0.156).

### Relationships between HER2 IHC positive (scored 3+) rate and the number of tumor containing fragments

**Fig. 2:**
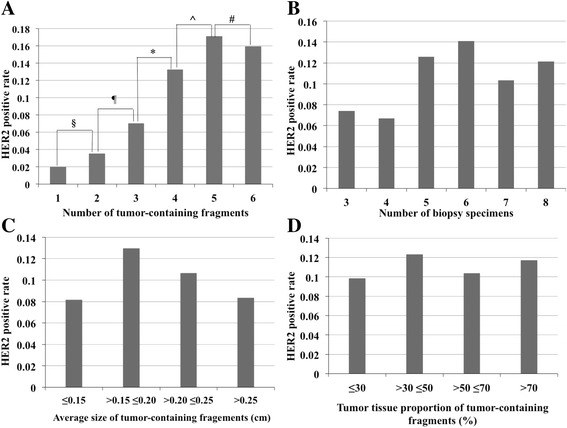
The associations of HER2 IHC positive (scored 3+) rate with tumor-containing fragment number, biopsy specimen number, average size and tumor tissue proportion of tumor fragments. **a** The HER2 IHC positive (scored 3+) rate exhibited elevation with increased tumor fragment numbers (*P* = 0.004). The rates of 1, 2, 3, 4, 5 and 6 fragments were 2.0, 3.5, 7.0, 13.2, 17.1, and 15.9%. The 4-fragment subgroup showed a much higher HER2 IHC positive rate than the 3-fragment subgroup. No statistical difference is reached among subgroups with 4, 5 and 6 fragments. ^§^ *P* = 0.6, ^¶^ *P* = 0.263, *P = 0.035, ^*P* = 0.299, ^#^ *P* = 0.812. **b** HER2 IHC positive (scored 3+) rates were 7.4, 6.7, 12.6, 14.1, 10.3, and 12.1% when the biopsy number was 3, 4, 5, 6, 7, and 8 respectively without significant differences (*P* = 0.127). **c** HER2 3+ rates of the subgroups divided based on average size of tumor fragments were 8.2, 12.9, 10.7 and 8.3% without statistical difference (*P* = 0.397). **d** HER2 3+ rates of subgroups divided based on tumor tissue proportion of tumor fragments were 9.8, 12.3, 10.4 and 11.7% (*P* = 0.825)

HER2 IHC positive (scored 3+) rates were 2.0, 3.5, 7.0, 13.2, 17.1, and 15.9% when the tumor fragment numbers were 1, 2, 3, 4, 5 and 6, respectively. The rate demonstrated an elevating pattern with the increase of tumor-containing fragment number (*P* = 0.004) (Table 4, Fig. 2a). No significant differences were found in HER2 IHC positivity either between the subgroups with 1 and 2 tumor fragments (*P* = 0.6) or between those with 2 and 3 tumor fragments (*P* = 0.263). The 4-fragment subgroup showed a significantly higher HER2 IHC positive rate than the 3-fragment subgroup (13.2% *vs* 7.0%, *P* = 0.035). After the fragment number reached 4, although slightly elevated HER2 IHC positive rates were observed with further increase of fragment number (17.1% of the 5-fragment subgroup and 15.9% of the 6-fragment subgroup), no statistical significance was reached (4 *vs* 5-fragment subgroup, *P* = 0.299; 5 *vs* 6-fragment subgroup, *P* = 0.812).

**Table 4:** Comparison of HER2 IHC positivity based on tumor-containing fragment number, biopsy specimen number, average size and tumor tissue proportion of tumor-containing fragments

	Total	HER2		*P* value
		3+, n (%)	Non-3+, n (%)	
Total	890	100 (11.2)	790 (88.8)	
Number of tumor-containing fragments				0.004*
1	51	1 (2.0)	50 (98.0)	
2	85	3 (3.5)	82 (96.5)	
3	171	12 (7.0)	159 (93.0)	
4	348	46 (13.2)	302 (86.8)	
5	117	20 (17.1)	97 (82.9)	
6	113	18 (15.9)	95 (84.1)	
8	4	0 (0)	4 (100)	
10	1	0 (0)	1 (100)	
Number of biopsy specimens				0.127
1	51	0 (0)	7 (100)	
2	85	0 (0)	20 (100)	
3	171	2 (7.4)	25 (92.6)	
4	348	14 (6.7)	195 (93.3)	
5	117	16 (12.6)	111 (87.4)	
6	113	61 (14.1)	372 (85.9)	
7	0	3 (10.3)	26 (89.7)	
8	4	4 (12.1)	29 (87.9)	
10	1	0 (0)	5 (100)	
Average size of tumor fragements (cm)				0.397
≤0.15	110	9 (8.2)	101 (91.8)	
>0.15 ≤ 0.20	417	54 (12.9)	363 (87.1)	
>0.20 ≤ 0.25	291	31 (10.7)	260 (89.3)	
>0.25	72	6 (8.3)	66 (91.7)	
Tumor tissue proportion of tumor fragments (%)				0.825
≤30	132	13 (9.8)	119 (90.2)	
>30 ≤ 50	324	40 (12.3)	284 (87.7)	
>50 ≤ 70	289	30 (10.4)	259 (89.6)	
>70	145	17 (11.7)	128 (88.3)	

“n” refers to the number of cases

*P <0.05

Since when the tumor fragment number was greater than or equal to 4 (≥4), the elevation of HER2 IHC positive rate showed no statistical difference, 4 was regarded as a suitable cutoff value. Comparison of HER2 IHC positive (scored 3+) rate was then made between the cases with less than 4 (<4) tumor fragments and ≥4 tumor fragments. Not surprisingly, the rate of cases with ≥4 tumor fragments (14.4%) was significantly higher than that with <4 fragments (5.2%) (*P* < 0.001).

### The relationships between HER2 IHC positive (scored 3+) rate and the number of biopsy specimens

We made comparisons of HER2 IHC positive (scored 3+) rates among subgroups divided based on the number of biopsy specimens. The rates were 7.4, 6.7, 12.6, 14.1, 10.3, and 12.1% when the numbers were 3, 4, 5, 6, 7, and 8, respectively. The assessment showed that HER2 IHC positive (scored 3+) rate did not exhibit significant differences among these subgroups (*P* = 0.127), indicating that the HER2 IHC positive rate was not associated with biopsy specimen number directly (Table 4, Fig. 2b).

### The associations of HER2 IHC positive (scored 3+) rate with the average size and tumor tissue proportion of tumor fragments

Besides tumor fragment number and biopsy specimen number, the size of tumor fragments and tumor tissue proportion may also potentially affect HER2 results. To explore the influence of tumor fragment size on HER2 IHC positivity, we compared the positive rates among subgroups divided based on the average size of tumor fragments. Based on the size, the specimens were divided into 4 subgroups: ≤0.15 cm, >0.15 ≤ 0.20 cm, >0.20 ≤ 0.25 cm, and >0.25 cm. HER2 3+ rates of the subgroups were 8.2, 12.9, 10.7 and 8.3% respectively. No significant difference was identified among these subgroups (*P* = 0.397) (Table 4, Fig. 2c).

Based on the proportion of tumor tissue, the specimens were divided into 4 subgroups: ≤30, >30 ≤50, >50 ≤70, and >70%. The HER2 IHC positive rates of these subgroups were 9.8, 12.3, 10.4 and 11.7% respectively. The differences did not reach statistical significance (*P* = 0.825) (Table 4, Fig. 2d).

### Comparison of HER2 status in the paired biopsy and resected specimens

The HER2 status of 459 paired biopsy and surgical specimens was evaluated (Table 5). In biopsy specimens, there were 52 HER2 3+ cases (11.3%), 138 HER2 2+ cases (30.1%) and 269 HER2 0/1+ cases (58.7%). In resected specimens, 45 cases (9.8%) were HER2 IHC positive (scored 3+), 119 cases (25.9%) were HER2 equivocal (scored 2+), 295 cases (64.2%) were HER2 IHC negative (scored 0/1+). The overall concordance rate between the biopsy and resected specimens was 71.5% (328 cases).

**Table 5:** IHC status of HER2 in paired biopsy and resected specimens

Biopsy specimens	Resected specimens				
	0	1+	2+	3+	Total
0	47	54	20	1	122
1+	33	92	19	3	147
2+	17	40	71	10	138
3+	1	11	9	31	52
Total	98	197	119	45	459

Among the 459 patients, there were 21 cases with 1 tumor fragment (4.6%), 40 cases with 2 tumor fragments (8.7%), 89 cases with 3 tumor fragments (19.4%), 183 cases with 4 tumor fragments (39.9%), 68 patients with 5 tumor fragments (14.8%), 55 cases with 6 fragments (12.0%), 3 cases with 8 tumor fragments (0.7%).

### The predictability of HER2 IHC positivity in relation to tumor-containing fragment number

**Fig. 3:**
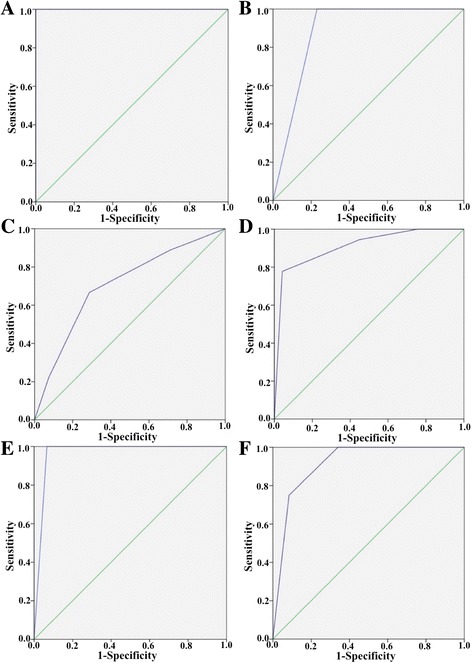
ROC curve analysis of the positive predictability of biopsy specimens. **a** 1 tumor-containing fragment. AUC = 1, *P* = 0.099. **b** 2 tumor-containing fragment. AUC = 0.885, *P* = 0.194. **c** 3 tumor-containing fragment. AUC = 0.705, *P* = 0.045. **d** 4 tumor-containing fragment. AUC = 0.909, *P* = 0.039. **e** 5 tumor-containing fragment. AUC = 0.978, *P* < 0.001. **f** 5 tumor-containing fragment. AUC = 0.915, *P* < 0.001

ROC curves were constructed and compared to evaluate the predictability of HER2 IHC positivity (positive predictability) based on the tumor fragment number (Fig. 3). When the fragment number was 1 or 2, biopsy specimens cannot predicted HER2 status in the surgical specimens reliably (AUC = 1, *P* = 0.099 and AUC = 0.885, *P* = 0.194) (Fig. 3a, b). When fragment number reached 3, biopsy specimens started to show predictability of HER2 positivity. The AUC values of 3, 4, 5, and 6 fragments were 0.705 (*P* = 0.045), 0.909 (*P* = 0.039), 0.978 (*P* < 0.001), and 0.915 (*P* < 0.001) respectively (Fig. 3c, d, e, f). Z test was performed to compared the AUC values of the ROC curves. It turned out that 4, 5 and 6 fragments showed better performance than 3 fragments in the positive predictability (*P* < 0.05). However, no significant differences were found among 4, 5 and 6 fragments (*P* > 0.05). Thus, we adopted 4 fragments as an appropriate cutoff of the positive predictability.

## Discussion

Assessment of HER2 in biopsy specimens is as important as in surgical specimens, because many GC patients are at advanced stage with inoperable lesions, besides, trastuzumab might become a promising future neoadjuvant regimen and there is an ongoing clinical trial in Japan to explore the feasibility [19]. As shown in several previous studies, concordance rate of HER2 status between biopsy and surgical resected specimens is fairly high (ranging from 74.1% to 96.1%) [20–22]. However, heterogeneity may result in discrepant HER2 results between the biopsy and resection specimens [2, 20, 21, 23], and usually leads to false negative results in biopsy specimens [22]. The mechanisms leading to the heterogeneity are still largely unknown but possibilities include neoplastic clones in which HER2 is amplified in an otherwise HER2 negative tumor or silencing of HER2 expression in an area of a tumor with homogeneous HER2 amplification [24]. To cope with the issue, we explored how HER2 IHC positive (scored 3+) rate would be affected by tumor-containing fragment number, biopsy number, average size and tumor tissue proportion of tumor fragments.

We found that among the above mentioned 4 factors related to sample amount, only tumor fragment number significantly affected HER2 IHC positive (scored 3+) rate. The rate elevated with the increase of tumor fragment number. The positivity reached a relative high level when the number reached 4 (13.2%, *P* = 0.035 *vs* 3 fragments). After that, HER2 IHC positive level slightly elevated with further increase of fragment number but without statistic significance. By comparing the paired biopsy and surgical specimens, we figured out that biopsy specimen exhibited predictability of HER2 positivity when fragment number reached 3 (AUC = 0.705, *P* = 0.045). However, 4, 5 and 6 fragments showed better performance (*P* < 0.05).

Visually, 5 tumor fragments gave the highest HER2 IHC positivity (17.1%) and the best performance in the positive predictability (AUC = 0.978, *P* < 0.001), and seemed to be a natural break at fragment numbers. However, lack of statistical differences when compared with 4 and 6 fragments did not support itself as an appropriate cutoff value. Thus, we adopted 4 as a suitable cutoff, and it is reasonable to conclude that ≥4 tumor fragments should be obtained to get a reliable HER2 result.

Compared with other recent studies, the current one was consistent with the study of Ahn et al. [11]. In that study, 702 paired biopsy and gastrectomy specimens were analyzed, they concluded that 4 fragments (at least) be recommended to minimize the differences in HER2 scores between biopsy and resection specimens [11]. Compared with that research, our data derived from direct comparisons of HER2 IHC positive (scored 3+) rates and predictability based on tumor fragment number. The consistency of the results further confirmed our conclusions.

Our findings were not in line with the studies of Gullo et al. [12] and Tominaga et al. [13]. In the research of Gullo et al., a minimum of 5 samples was identified as the most accurate in predicting HER2 status [12]. Tominaga et al. found that five tumor-containing biopsy specimens from the proximal part of the tumor reliably determine HER2 status in GC [13]. Unlike this research, both of the two studies adopted virtual biopsy for HER2 assessment, which was not an actual reflection of routine clinical practice.

Biopsy number, unlike tumor fragment number, did not influence HER2 IHC positive rate significantly (*P* = 0.127). Multiple biopsies are often recommended to ameliorate diagnostic accuracy in HER2 evaluation in biopsy specimens [8–10]. In these recommendations, the biopsy specimen number rather than the tumor-containing fragment number was rendered. According to the current study, tumor fragment number showed more important values for HER2 IHC evaluation in biopsy specimens of GC. It might not be practical to determine precise tumor fragment number during performing a biopsy, however, more attentions are required to the number in developing guidelines for HER2 assessment in GC in the future.

In this study, we mainly focus on HER2 IHC status. IHC was chosen for HER2 assessment because it is reliable, easy to perform and therefore, used more often. In routine clinical practice, for HER2 2+ cases, FISH or SISH is needed to determine HER2 status. It would be meaningful to compare HER2 status between biopsy and resected specimens by FISH/SISH in HER2 2+ patients in future studies.

Based on the current study, interpretation of HER2 IHC results should be with caution in biopsy specimens of GC. It is recommendable that the numbers of tumor-containing fragments be described in HER2 IHC pathology report for clinical reference. If a biopsy specimen with less than 4 tumor fragments shows negative HER2 IHC result, it is reasonable to perform a repeated biopsy to obtain more tumor tissue for HER2 test to get a more accurate result, especially for those inoperable patients with indications for trastuzumab treatment.

## Conclusion

Tumor-containing fragment number affects HER2 IHC positive (scored 3+) rate in endoscopic biopsy specimens. Greater than or equal to 4 (≥4) tumor fragments give better HER2 IHC positivity and better performance in predicting HER2 status of resected specimens. We recommend that tumor fragment number be described in the pathology reports for clinical reference. The oncologists will be aware that for those HER2 IHC negative patients with less than 4 tumor fragments, additional biopsies might be needed for further HER2 analysis to avoid missing eligible patients for the molecular-targeted treatment.

## Abbreviations

FISH: Fluorescence in situ hybridization
GC: Gastric cancer
HER2: Human epidermal growth factor receptor 2
IHC: Immunohistochemistry
SISH: Silver in situ hybridization
